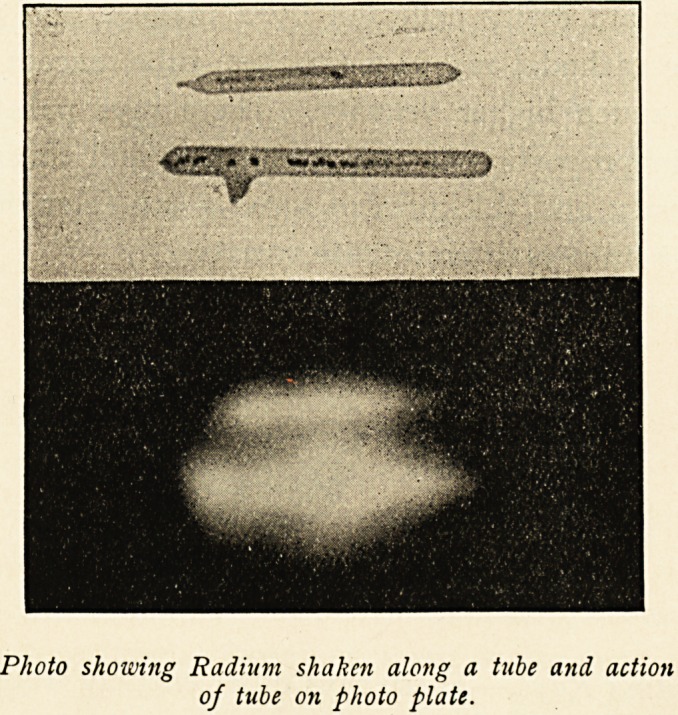# Radium and Some of Its Physical and Therapeutic Properties

**Published:** 1910-03

**Authors:** James Mackenzie Davidson


*"Scire est nescire, nisi id me*
*Scire alius sciret."*

MARCH, 1910.

INDEX THROUGH

### RADIUM AND SOME OF ITS PHYSICAL AND THERAPEUTIC PROPERTIES.

BY

JAMES MACKENZIE DAVIDSON, M.B., C.M.

IN 1895 the discovery of X-rays by Röntgen led others to wonder if invisible radiations of a similar nature could be produced by other means than a Crookes' tube. In the following year Monsieur Henri Becquerel, in Paris, experimented in this direction, and by good fortune he first tried a salt of uranium. He took a photographic plate and wrapped it in black paper, which completely shielded it from all light. Upon this he placed his uranium salt, and exposed this to light, his idea being that the fluoresence induced by the light in the uranium might give rise to invisible rays, which, like the X-rays, would pass through the paper and darken the photographic plate. He found that the photographic plate was blackened beneath the salt; but on further investigation, he found that this effect was produced equally well without the uranium being exposed to the light. In short, he discovered that the property of emitting these invisible rays was a property inherent in the element uranium itself. He further discovered that these rays from uranium had the power, like X-rays, of discharging electrified bodies. These discoveries of Becquerel were, in effect, the discovery of what we now know as radio-activity. The term *radio-active* is applied to elements which spontaneously emit invisible rays, that possess the power of passing through opaque substances, which rays have likewise the power of discharging electrified bodies, of acting on a photographic plate, and of causing certain substances to fluoresce. They cause air and other gases to become temporarily conductors of electricity; lastly, they generate heat spontaneously.

Amongst other consequences these discoveries set Madame Curie investigating the radio-activity of various substances. She soon found that the mineral pitchblende was more radio-active than the uranium it contained. The inference was obvious, namely that the pitchblende must contain some substance more radio-active than uranium. Madame Curie therefore set herself to separate by chemical processes the various active substances in a large quantity of pitchblende residues. With the aid of her late distinguished husband, she succeeded firstly in separating a highly radio-active substance, which she called polonium (in compliment to her own nationality). Afterwards she succeeded in separating a substance which she called radium, which proved to be two million times more radio-active than a corresponding amount of uranium.

Up to this period most of us had been taught, and we believed, that what we call matter is composed ultimately of atoms, such atoms being indivisible entities. But since then the work of Sir J. J. Thompson, Rutherford, Sir William Ramsay, and Soddy has clearly proved that the atom of radium is disintegrating, and, moreover, that it is disintegrating spontaneously at a steady rate, which cannot be either accelerated or retarded by any known means.

It is precisely the radiant energy evolved by this disintegration which is of value to us in medicine. The quantity of radium which is obtainable is so small (being only one part in five millions of the best pitchblende) that it has never been experimented with as a drug to any extent, and its therapeutical properties in this respect are consequently unknown. But before saying anything more about its medical application, I will describe shortly this disintegration.

*Deviation of Rays in a strong magnetic field.*

The radium atom first gives off an atom of helium at a high velocity, about twenty thousand miles per second. This is called the alpha ray, and is beautifully illustrated by a little instrument devised by Sir William Crookes, and called the spintharoscope. The remainder of the atom evolves as a gas, which is called its emanation. This emanation in turn decomposes rapidly, losing half its energy in about four days. It also gives off an alpha particle, and after two decompositions finally results in radium C, which gives off three radiations, known as the alpha, beta, and gamma rays.

It is these three different kinds of rays that are important in the therapeutic effects of radium. But before proceeding to give an account of the effect of these rays upon certain diseases, I will give some detals as to their physical properties.

Radium disintegration

The alpha rays, as already mentioned, consist of atoms of the apparent mass of twice that of a hydrogen atom. They have been proved to be atoms of helium, travelling at the high velocity of twenty thousand miles per second. Their power of penetrating matter is small, and a sheet of ordinary writing paper is sufficient to stop them. They represent 80 per cent. of the total energy given out by the radium. They have great power in ionising gases, that is, in rendering gases conductors of electricity. When radium is contained in small sealed, glass tubes none of these alpha rays get outside ; and even when radium is spread upon metal plates, and kept fixed by a very thin varnish, even so only a small proportion of these rays are able to pass through the varnish. It thus happens that most of the medical effects of radium are the result of the beta and gamma rays. To sum up, the alpha rays affect a photographic plate, ionise gases, produce brilliant fluoresence on a sulphide of zinc screen, and are deflected to a very slight extent by a powerful magnet in a direction which shows them to carry a positive charge, and they represent 80 per cent. of the total disintegration of the radium atom. Being so easily stopped by a thin layer of matter, it was to be expected that their action on the skin would be powerful.

The next rays are called the beta rays. These are also material particles which have been called corpuscles. Their mass is one thousand times less than the size of an atom of hydrogen. They are negatively charged, travel at varying velocities, from half the velocity of light up to nearly the velocity of light. They are much more penetrative than the alpha rays, and it requires one centimetre thickness of lead to stop all of them. They are easily deflected by a magnet, and indeed are just like the cathodal ray in a Crookes' tube, except that they travel at a much higher velocity. These rays penetrate glass and metal in proportion to their velocities, and consequently a varying density and thickness of screens can filter out the less penetrative beta rays in an infinite variety.

*Relative sizes Alpha and Beta Rays.*

*The Gamma Rays.*—These rays differ entirely from the alpha and beta rays already described in being no doubt a wave disturbance in the universal ether ; they do not consist of projected material particles. They are apparently exactly the same as the X-rays of a Crookes' tube, except that they are much more penetrating. No X-ray can be produced, even with the highest vacuum tube, to approach the gamma rays in penetrability. Like the X-rays, they are not deflected by the most powerful magnet ; they are not refracted, nor do they suffer any diffraction. Like the X-rays, their wave length is unknown.

Another remarkable fact accompanying the disintegration of radium is the emission of heat. The chemical reaction which evolves the largest amount of heat is the union of hydrogen and oxygen to produce water. Radium in its transformation gives out four million times more heat than is given out by the explosive combination of a similar quantity of oxygen and hydrogen. All these rays from radium act upon a photographic plate and discharge electrified bodies ; they also act upon living cells destructively if exposure is sufficiently prolonged. They will cause diamonds to shine in the dark (although paste will not shine), and barium platinocyanide and willemite (a silicate of zinc), also fluoresce.

*Photo showing Radium shaken along a tube and action of tube on photo plate.*

Considering the before-mentioned properties of this remarkable substance, it seemed almost certain that it would prove of some use in medicine. Becquerel's accidental experience of a severe burn on his skin produced by carrying a tube of radium in his waistcoat pocket clearly demonstrated that it had some powerful physiological effects, and it only remained for the medical profession to discover in what cases such action would produce beneficial effects and in what manner radium could be best applied.

In April, 1903, I bought two small glass tubes, each said to contain five milligrammes of radium bromide. At that time I had no data to guide me as to what use it might be in medicine, nor how to apply it most effectively. It so happened that a patient at Charing Cross Hospital was brought to me from the Skin Department with a large rodent ulcer underneath the right lower eyelid. This ulcer had resisted X-ray and other treatment. It occurred to me to experiment upon it with radium; and as this was the first case ever treated with radium in Great Britain, I shall give a short description of the method adopted.

The ulcer measured one inch from above downwards, and three-quarters of an inch across. On May 21st, 1903, a tube with five milligrammes of radium bromide was applied for fifteen minutes to the upper border of the ulcer. The radium was first shaken down to the end of the tube, and then this end was applied directly to the part, and secured in position by a strip of rubber plaster attached to the healthy skin. On May 22nd the same tube was applied, for the same time, to another part of the ulcer, and on May 29th (that is eight days after the first application) some improvement was noticed in the parts treated. Treatment was continued over the various parts of the ulcer; on June 11th and 26th two tubes were applied, and the last application took place on July 10th, 1903. On December 7th the patient was quite cured; the skin became smooth and normal in appearance over the part, and it has remained so ever since, that is to say for nearly seven years. This result was very encouraging.

My next cure was that of a patch of tuberculosis verrucosa on the hand of a boy aged ten years. After four applications of about twenty-five minutes each time this was also cured.

The next case was one of recurrent carcinoma of the breast, where there were some small spots and nodules of recurrence. Of course the amount of radium used was small. At the same time, from the effect on the very small spots of recurrence the conclusion arrived at was that carcinoma cells were very resistent to radium, and that it was never likely to be a cure for this dire disease. This was in July, 1903. Again, in the same month, a case of rodent cancer in the right side of the nose was treated in a similar manner by these two tubes, and was completely cured.

Another case, treated in October, 1904, serves to illustrate the great utility of radium in rodent cancer of the eyelids. This patient was sent to me by Dr. Tempest Anderson of York. She had a rodent cancer at the inner canthus of the left eye, which had resisted previous treatment. Its situation rendered its removal a matter of great difficulty on account of its close proximity to the tear sac. On October 21st four tubes, each containing five milligrammes of radium, were applied to the ulcer for half an hour. On October 24th the same tubes were again applied for half an hour. On October 28th they were applied for twenty minutes. There had been some slight reaction, and improvement was noticed. On November 8th there was some reaction, and no further application was made at this time. On November 15th the induration was greatly diminished, but the parts were red and irritable. Five tubes were put on for twenty minutes. On November 30th much improvement was evident, and four tubes were applied for half an hour. By December 13th the ulcer was nearly well, only a small speck in the centre remaining, to which two tubes were then applied for half an hour. In the following year, on January 26th, and again on May 3rd, Dr. Tempest Anderson reported the case as perfectly cured.

The next case is of interest on account of the constitutional effects of the radium on the patient. On July 29th a case of rodent cancer of the face came under my care. It was a case of twenty-five years' standing, in which two operations had been performed, and a variety of treatments had been tried in vain. It was too extensive a case for me to hope to cure with the small amount of radium at my disposal. However, by going over the surface, in turn from place to place, quite a remarkable improvement resulted. Unfortunately I had to go abroad, and the treatment was discontinued for a time. On my return treatment was resumed vigorously, and this led to only a partial arrest of the disease. On two occasions a very severe reaction followed the application, which in many respects appeared like an attack of erysipelas. There was headache, sickness, high temperature, and a large area round the rodent was of a red colour. I sent some of the blood from the ulcer and some of the discharge to Dr. Bulloch, of the London Hospital, who reported it absolutely sterile. It is evident that in this case the symptoms were due to some absorption, the result of the radium application, possibly owing to the exposure being too prolonged. Later on, in the hope of arresting the progress of this disease, emanations of radium dissolved in water, supplied by Sir William Ramsay, were injected subcutaneously in the neighbourhood of the ulcer. The patient was also made to wear a pad of thorium hydroxide. All were of no avail, and she died eventually of deep extension of the disease. In this connection it may be remarked that further experience has shown that, in severe cases of malignant disease, injections of the emanation or swallowing it in large quantities has failed to produce any good effect.

Since that time I have treated a large number of cases with radium. Small epitheliomata of skin and tongue can be cured; moles, nævi, port wine stains, can be removed; and several small rodent ulcers have been cured by one application.

Quite recently I have found that radium cures dermatitis from X-ray burns. The method adopted is to place a tube with about twenty milligrammes of radium on to the part for ten minutes, and to renew this application the second day. Slight reaction usually begins about the fifth or sixth day, reaching its height on the eighth. A crust may then be formed, and the inflammatory process gradually subsides. In about six weeks either a great improvement or a cure results. How far these terrible cases of severe and extreme X-ray burn, of which you have doubtless heard, may be benefited by radium treatment I do not know, as the quantity of radium I possess is too small to be employed in such cases ; but from the excellent effect it has upon small lesions, it seems likely that with larger quantities, equally satisfactory results might be obtained in severe cases. However, one may hope that the adoption of proper protection by those working with X-rays will prevent these disastrous cases from ever occuring in future.

One more reference to my own work. In September, 1903, I first tried radium in a case of a granulation of the eyelids. This experiment did not meet with any success, which may have been due to the small quantity of radium then available, and to inexperience in its use. It was not until February, 1906, when Mr. Arnold Lawson sent me a case of spring catarrh of the eyelids that I made any further application of radium to diseases of the eye. This disease, which is not very common in this country, has been hitherto incurable. The patient was a boy, aged 13, who had suffered for eighteen months. Mr. Lawson had tried a variety of treatments for about a year without any effect. On February 9th the upper lid of the left eye was everted, and three radium tubes, containing altogether thirty-nine milligrammes of radium bromide, were held in contact with the granulations for fifteen minutes. On February 19th the treated eye was much better, except that, five days after the treatment, some reaction followed. On this occasion four tubes, containing forty-four milligrammes, were applied for ten minutes to the left everted eyelid, and for fourteen minutes to the right. It was noticed that the granulations of the left eyelid seemed better. On May 8th the right eye was much better, while the left had again improved a little. The progress towards recovery was uninterrupted after a few more treatments, and the patient got perfectly well, the lids being smooth and without any scar. This patient has remained perfectly well ever since, and the cure may now after four years be looked upon as permanent.

Three similar cases, one being of six years' standing and one an extremely severe case, have done exceedingly well with radium treatment, and all are now nearly cured.

In treating lesions of the eyelid with radium it is most important to protect the eyelashes by means of lead foil, otherwise they may be destroyed. It is also advisable in prolonged exposures generally to protect the normal part (on which you do not mean to act) by means of lead foil covered with thin rubber. Further, in the treatment of eye diseases, when the radium tube has to be held in position, it is most important to wrap the upper part of the tube in several folds of lead foil so as to protect the fingers of the person holding it. Otherwise severe burns may result if several cases are being treated consecutively.

With regard to diseases of the eye, I am carrying on researches at the Royal London Ophthalmic Hospital, Moorfields, assisted by Mr. Arnold Lawson. As to this it may suffice to say in the meantime that radium has yielded most astonishingly favourable results in many cases, some of which were reported in papers by myself and Mr. Arnold Lawson in *The Transactions of the Ophthalmological Society of the United Kingdom for* 1909. In cases of hypopyon ulcer of the cornea, corneal ulcers generally, episcleritis and cases of pterygium (even after several operations have failed to cure), it has yielded results superior to any other known method of treatment. Radium may therefore be looked upon as one of the most important therapeutic agents in superficial eye diseases.

In this connection it may be mentioned that if a tube of radium be held anywhere close to the eyes, in the dark, with the eyelids closed, a very peculiar sensation of diffused light is produced. This is in no way due to the visible rays, as the effect is quite the same if the tube of radium be wrapped in black paper. We have not found that the deeper diseases of the eye are benefited by radium rays ; but work is this direction as yet has been too limited to admit of our speaking with any certainty in the matter.

I will now pass from my own work with radium to that of others. With few exceptions all my cases have been treated with radium bromide enclosed in sealed glass tubes, and therefore the rays utilised were all the gamma rays, and most of the beta rays, but none of the alpha rays.

In Paris, where the Radium Institute could command large quantities of radium salts, Dr. Wickham and others were able to use it spread upon surfaces of metal of various sizes. They used for this purpose the sulphate of radium, more or less impure, because it was insoluble, unlike the bromide, which is very soluble and hygroscopic. Special varnish is necessary for spreading the radium, because having a powerfully destructive action upon organic substances, it would eventually cause any varnish to become brittle and friable, so that it might peel off and lead to loss of the radium. In a spread apparatus of this kind, if the coating of varnish be very thin, a few of the alpha rays can get through. It is usual to wrap round these apparatus a thin sheet of pure rubber, so as to protect it from contamination by the diseased surface to which it may be applied.

Dr. Dominici, of Paris, was the first to experiment with radium rays through metal screens of various thicknesses. As the beta rays are not uniform, but vary greatly in velocity, it follows that, by interposing screens of varying density and thickness, beta rays of different velocity can be allowed to impinge upon the diseased part. If lead or platinum screens of considerable thickness be interposed the beta rays of a very high velocity, and the gamma rays, are allowed to pass through. The various apparatus used by Messrs. Wickham and Degrais are described in a table in their large book entitled *Radiumtherapie.* The size of the surface of the apparatus is given, and the quantity of the radium salt spread upon it and its radio-activity. These apparatus may be used with thin rubber sheeting over them, or with screens of aluminium or lead interposed, and then the rubber covering.

The best manner in which to standardise each apparatus is to measure the radio-activity, and then to divide this by the surface. For example: If four square centimetres of surface had radium spread upon it of a total activity of a million units, taking an equal quantity of uranium as one, then, of course, each square centimetre would contain 250,000 units.

The question of dosage naturally at once presents itself. Having a given quantity of radium, what screen should be used, and how long should it be applied to the part to be treated? This is a matter of trial and experience, and as yet no very precise rules can be laid down. Indeed, there seems to be a very wide range, within which good results can be obtained without damage to the normal tissue. I will now describe some cases from Dr. Wickham's book, and the apparatus and method employed by him.

**Case 1.**—An epitheliomatous ulcer on the nose. On December 6th he applied for one hour an apparatus whose radio-activity was 16,100 per square centimetre. No screen was used, and upon a portion of the ulcer, not covered by the first apparatus, another apparatus of 30,000 units activity was applied for twenty-five minutes. The same treatment was adopted on the 7th and the 10th. Already the appearance of the ulcer was improved. Ten applications of the first apparatus were made on consecutive days for an hour at a time. On January 10th the reaction was at its height, and a yellow crust had formed over its whole surface, which may be called the "Radium Crust." On pressing the crust a few drops of sero-purulent discharge exuded. On January 25th the crust spontaneously came away, leaving a surface of skin of normal appearance. Dr. Wickham remarks that he thinks the dosage in this case was too intense, as six or seven hours' application would have been sufficient.

The second case I will refer to is that of a baby with an angioma of the lower lip. Two apparatus, one of 17,700 units activity per square centimetre and the other 16,100 units, were placed one above and one below the small tumour. Interposed between them was a thin sheet of aluminium, .02 millimetres thick, and the whole was covered with thin india-rubber sheeting. Six applications of twenty minutes each were made on alternate days during a month; the whole of the treatment was complete in eight months, and fifteen months afterwards the lip presented a normal appearance, as can be seen in the slide.

The next slide is that of an epithelioma of the ear. Apparatus of 16,100 units was applied without any screen, six applications of one hour each being made for six days consecutively. Severe reaction followed. Fifteen months afterwards a suspicious spot was observed, and this was treated for one hour with an apparatus of 26,300 units. A complete cure was the result.

Another case of epithelioma may be cited to show that good results may be obtained without any severe reactionary irritation. If cotton wool or wadding to a total thickness of one centimetre be interposed between the apparatus and the parts to be treated, long exposures can be given, and equally good results obtained.

The next case is one of extensive dark-coloured nævus of the face. An apparatus of 20,500 units activity was applied, with cotton wool wadding interposed, about one centimetre thick. Applications for one hour were made every alternate day for five days. The reaction depended upon the thickness of the interposed wadding. Finally a severe reaction was produced, and after various applications a very satisfactory result was obtained a year after the cessation of the treatment.

The next is a case of a large tumour of the parotid region in a man aged 54. In this case the apparatus was covered with lead one or two millimetres thick, so that only the more penetrative beta and gamma rays were utilised. The average radio-activity of the apparatus employed was 13,000 units. This apparatus was left on the whole night, and the next night was placed upon a fresh region. In this manner surface irritation was avoided, and deep action of the rays secured. In addition to this an apparatus was placed, without a screen, upon the ulcerated surface at the apex of the tumour. Besides this, on four occasions, a tube of thin gold, containing five milligrammes of radium, was buried in the centre of the tumour, so that the tumour was traversed by rays from within and without. The tube was left in for twenty-four hours, and the final result is seen on the adjacent slide. Although there was some deep thickening yet to be felt, the improvement is certainly very remarkable.

In the treatment of larger tumours, such as carcinoma, radium ought to be filtered through two or three centimetres of lead covered with rubber sheeting, and between this and the skin half a dozen or so layers of paper, or two folds of lint, should be interposed, so as to avoid superficial irritation of the skin. It is found that the metal screen alone gives off from its under surface under the action of the radium rays passing through it secondary rays, which are very easily absorbed ; and if left directly in contact with the skin, these secondary rays would produce irritation and burning. Apparatus of this kind, according to the radio-activity, can be applied during the whole night, and continued to a total of 60 to 120 or more hours, and then the effect watched for a month, and if no undue reaction follows, another application may be made. The result of the treatment of large tumours, whether internal or external, if carcinomatous, is unsatisfactory. If there is ulceration to begin with, the ulcers often heal up under radium treatment, and this is apt to raise hopes in the patient's mind of a possible cure, which is not likely to be realised. But it is claimed that radium relieves pain in these cases, and may to some extent delay growth.

When at the Radium Institute in Paris I saw drawings of an interesting case treated by Dr. Dominici. Into the centre of what was believed, after microscopical examination, to be a large round-cell sarcoma of the neck, he introduced a glass tube containing ten milligrammes of radium, which was enclosed in a silver tube half a millimetre thick, which again was surrounded by gauze. This he left *in situ* for forty-eight hours continuously, and this led to complete disappearance of the tumour. I saw drawings of the patient both before and after the treatment.

Also when in Paris I saw photographs of a patient of Dr. Claude's, whose hands and fingers were bent and stiff from chronic rheumatism. These hands were treated by pads of radio-active mud, with the result that the patient was enabled to straighten his fingers and there was considerable improvement in the general condition of the hands.

Altogether the work with radium which has been done in Paris by Dr. Wickham, Dr. Dominici, and others has greatly extended our knowledge of radium-therapy.

Professor Nagelschmidt, in an article in the *Klinisch-Therapeutische Wochenschrift*, Vienna, 1909, comes to the conclusion that radium is not effective in cases of visceral tumours or of the larger cutaneous tumours. Moreover, such cases in which it has been introduced into the cavities of the body, the uterus, rectum, or the nose, and is said to have produced a rapid improvement, must be looked upon with a certain amount of scepticism. He also refers in his article to treatment by means of radium emanations dissolved in water. He advises the drinking cure to be carried out by beginning with small doses :

the first day 2,000, second day 3,000, and the third day 5,000 emanation units. After that, from day to day, an increase of 5,000 up to 30,000 units, which remains the dose until the end of the cure, when there is a gradual decrease of 10,000 units per day down to nothing. The cure lasts from twenty-five to thirty days of continuous treatment, The patient must be warned that very often, in fact almost in every case, immediately after the first dosage, a so-called reaction will set in, which means a more or less considerable increase of pain and discomfort. It often happens that patients suffering from acute symptoms in any joint complain of violent pain in the other joints as well which may have been previously affected or have been cured. Considerable swelling and inflammation may supervene, so that patients may have to be kept in bed a day or two.

Another form of radium therapy consists in radium emanation baths. As the skin does not absorb gases, there is no doubt that whatever efficiency these baths have is due to inhalation of the emanations set free. Of course, radium emanation must be used within twenty-four hours of being taken from the apparatus which supplies it, as once it is removed it rapidly deteriorates, and is only half strength in about four days. Dr. Nagelschmidt suggests that drinking emanations of radium may give rise to a transient albuminuria. Dr. Lowenthal comes to the conclusion that cases of chronic rheumatism and of chronic neuritis give indications of being suitable for radium treatment. As an instance of the deleterious effects of an overdose of emanations, he mentions that one of the great Berlin High School teachers was occupied in investigating a substance containing radium. It was 240 grammes of a by-product, which had remained for years in a room, and had developed therein enormous masses of emanations. While dealing with this substance symptoms of increasing physical discomfort presented themselves, which became so aggravated that the savant was obliged to take to his bed. The doctor found a very high percentage of albumen in the urine, and he failed to diagnose the illness. All the symptoms disappeared eight days afterwards, and the patient completely recovered.

Dr. Nahmmacher[1] gives particulars of sixty patients treated by him. Among the successful cases were small recurrent nodules after cacinoma of the breast, two cases of lupus, one of cutaneous tuberculosis, warts, pigmented spots on the skin, scars and herpes, angioma, telangiectasis, and a case of nævus vascularis extending over the left half of the face. The treatment proved ineffectual in cases of frost-bitten hands, myoma, advanced inoperable carcinoma, and deep-seated tumours. Nevertheless, in many of these cases it relieved the pain. In a remarkable case of carcinoma of the cervix he inserted a tube with ten milligrammes for five weeks. This seemed to cure condition completely, but patient died from recurrence in one of her ovaries.

Some good work has been done with radium by Dr. Williams, of Boston, and Dr. Abbé, of New York. The latter has come to the conclusion that " radium ranks not with caustics, cautery or medication, but with specifics." This does not mean, he is careful to add, that radium is a specific for cancer in the popular sense, but for erratic cell growths constituting some types of tumour tissue in the earlier stage of invasion or of moderate development.

I trust that I have been able to give you some insight into the physical properties and medical uses of radium. It is unfortunate that at the outset undue hopes were raised that it would be a cure for cancer ; and although at present we cannot hope that it will cure large malignant tumours, yet the results obtained in other directions are so remarkable that workers should persevere in their endeavour to ascertain what further results may be obtained. It is to be hoped that the Radium Institute which will be opened shortly in London will carry on investigations, with a large quantity of radium at its command, which may lead to great and unforeseen developments of its use in medicine.

The lecture was illustrated by an electroscope devised by the lecturer, by a radium clock which had been going for six years (kindly lent by Dr. Martindale), and a number of lantern slides, some to show the physical properties of radium, and some (coloured slides) taken from the pictures in Wickham and Degrais' *Radiumtherapie*, to show the condition, before and after treatment, of patients suffering from a variety of diseases.

[1] *München. Med. Wchnschr.*, 1908, lv. 140.

## Figures and Tables

**Figure f1:**
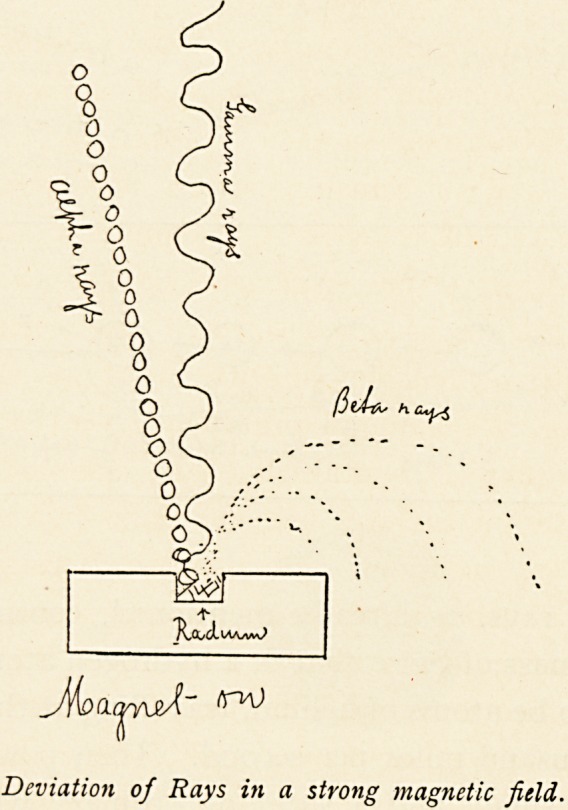


**Figure f2:**
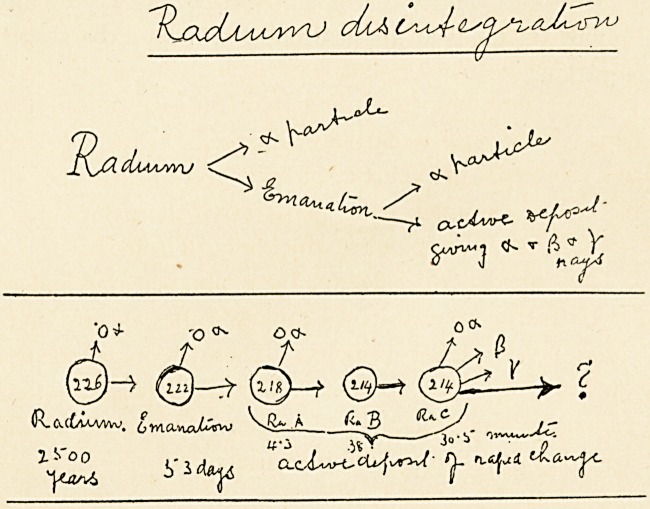


**Figure f3:**
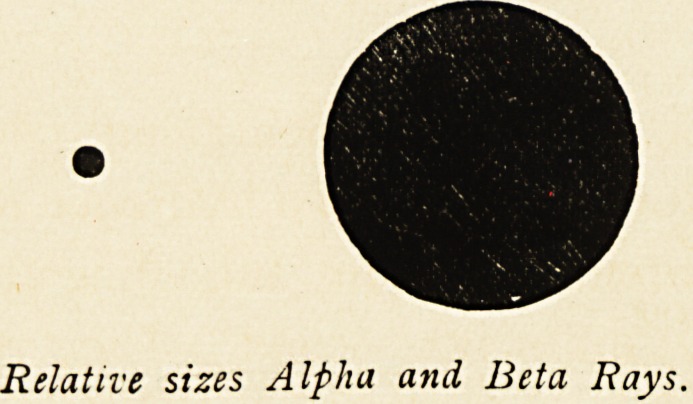


**Figure f4:**